# Multi-Institutional Dosimetric Evaluation of Modern Day Stereotactic Radiosurgery (SRS) Treatment Options for Multiple Brain Metastases

**DOI:** 10.3389/fonc.2019.00483

**Published:** 2019-06-07

**Authors:** Irina Vergalasova, Haisong Liu, Michelle Alonso-Basanta, Lei Dong, Jun Li, Ke Nie, Wenyin Shi, Boon-Keng Kevin Teo, Yan Yu, Ning Jeff Yue, Wei Zou, Taoran Li

**Affiliations:** ^1^Department of Radiation Oncology, Rutgers Cancer Institute of New Jersey, Rutgers University, New Brunswick, NJ, United States; ^2^Department of Radiation Oncology, Thomas Jefferson University Kimmel Cancer Center, Philadelphia, PA, United States; ^3^Department of Radiation Oncology, University of Pennsylvania Abramson Cancer Center, Philadelphia, PA, United States

**Keywords:** stereotactic radiosurgery, multiple brain metastases, conformity index, dynamic conformal arcs, volumetric arc therapy

## Abstract

**Purpose/Objectives:** There are several popular treatment options currently available for stereotactic radiosurgery (SRS) of multiple brain metastases: ^60^Co sources and cone collimators around a spherical geometry (GammaKnife), multi-aperture dynamic conformal arcs on a linac (BrainLab Elements™ v1.5), and volumetric arc therapy on a linac (VMAT) calculated with either the conventional optimizer or with the Varian HyperArc™ solution. This study aimed to dosimetrically compare and evaluate the differences among these treatment options in terms of dose conformity to the tumor as well as dose sparing to the surrounding normal tissues.

**Methods and Materials:** Sixteen patients and a total of 112 metastases were analyzed. Five plans were generated per patient: GammaKnife, Elements, HyperArc-VMAT, and two Manual-VMAT plans to evaluate different treatment planning styles. Manual-VMAT plans were generated by different institutions according to their own clinical planning standards. The following dosimetric parameters were extracted: RTOG and Paddick conformity indices, gradient index, total volume of brain receiving 12Gy, 6Gy, and 3Gy, and maximum doses to surrounding organs. The Wilcoxon signed rank test was applied to evaluate statistically significant differences (*p* < 0.05).

**Results:** For targets ≤ 1 cm, GammaKnife, HyperArc-VMAT and both Manual-VMAT plans achieved comparable conformity indices, all superior to Elements. However, GammaKnife resulted in the lowest gradient indices at these target sizes. HyperArc-VMAT performed similarly to GammaKnife for V_12Gy_ parameters. For targets ≥ 1 cm, HyperArc-VMAT and Manual-VMAT plans resulted in superior conformity vs. GammaKnife and Elements. All SRS plans achieved clinically acceptable organs-at-risk dose constraints. Beam-on times were significantly longer for GammaKnife. Manual-VMAT_A_ and Elements resulted in shorter delivery times relative to Manual-VMAT_B_ and HyperArc-VMAT.

**Conclusion:** The study revealed that Manual-VMAT and HyperArc-VMAT are capable of achieving similar low dose brain spillage and conformity as GammaKnife, while significantly minimizing beam-on time. For targets smaller than 1 cm in diameter, GammaKnife still resulted in superior gradient indices. The quality of the two sets of Manual-VMAT plans varied greatly based on planner and optimization constraint settings, whereas HyperArc-VMAT performed dosimetrically superior to the two Manual-VMAT plans.

## Introduction

Stereotactic radiosurgery (SRS) was first conceptually introduced by neurosurgeon, Lars Leksell, in 1951 (1, 2). The evolution of this technology alongside advances in image guidance have enabled the Gamma Knife to serve as the leading workhorse for treating cranial malignancies with hypofractionation. Although it was the first of its kind to perform SRS, the Gamma Knife has not been the only player, with other accelerator modalities adapting to offer solutions for patients requiring SRS (3, 4). Advancements in hardware and software design have since propelled linacs to become a popular and more widely available technology for stereotactic treatment capability. This is particularly pertinent for the treatment of multiple brain metastases, which were traditionally treated with surgery and/or whole brain radiation therapy (WBRT).

With more studies promoting the benefits of SRS for multiple brain metastases such as: improved local control when adding SRS to WBRT (5–8), similar survival (WBRT+SRS vs. SRS only) (8–17) and less cognitive deterioration (SRS only) (18–21), the ratio of patients receiving SRS treatments annually increased 15.8 percentage points from 2004 to 2014 and the number of facilities offering SRS annually increased 19.2 percentage points (22). Supporting evidence for SRS of a large number of brain metastases has further contributed to this effect (14, 20, 23–29). This growing demand for SRS, coupled with the ease of access to conventional linacs, has stimulated the development of a number of new technologies to facilitate the implementation of linac-based SRS for the treatment of multiple metastases. The common goal of all these linac SRS techniques is to use a single isocenter to treat all of the metastases simultaneously, in order to avoid prohibitively long treatments with multiple isocenters and thereby improve patient comfort and throughput. The most current single isocenter linac-based SRS options include multi-aperture dynamic conformal arcs on a linac (30–32) (BrainLab Elements™ v1.5, Munich, Germany), volumetric arc therapy (VMAT) calculated with the conventional optimizer (33–43) (Varian Medical Systems, Palo Alto, CA) or VMAT delivery calculated with the newer Varian HyperArc solution (44–47).

With this large variety of commercially available SRS treatment techniques, it is important to assess and be aware of the different strengths and weaknesses of the numerous options available for patients seeking treatment for multiple metastases. As the different technologies have emerged, there have been a number of studies comparing some of the techniques against each other. Thomas et al. (48), Liu et al. (49), and Potrebko et al. (50) each compared VMAT to GammaKnife for 28 patients with 2–9 targets, 6 patients with 3–4 metastases and 12 patients with at least 7 metastases, respectively. Mori et al. compared Elements to GammaKnife for two patients each with 9 metastases (32). Ohira et al. (44) compared HyperArc to conventional VMAT for 23 patients with 1–4 metastases, meanwhile Slosarek et al. (46) has most recently compared CyberKnife, VMAT and HyperArc for a set of 15 patients with 3–8 metastases each. Overall, these studies have found that VMAT is generally comparable to GammaKnife (with some minor differences such as improved conformity indices at the cost of potentially increased low dose spread), as is Elements to GammaKnife, and similarly now HyperArc is to VMAT. However, most of the published studies have only compared two technologies to each other, with the exception of Slosarek et al. (46), which added CyberKnife to the mix. This makes it difficult to assess whether one technique may truly be superior to another for a certain patient scenario because there is a lack of comparison data on the same subset of patients for the multiple SRS techniques available. It is therefore the aim of this work to provide a more rigorous and comprehensive evaluation of the dosimetric differences between the following state-of-the-art SRS modalities: GammaKnife, Elements, Manual-VMAT, and HyperArc-VMAT.

## Methods and Materials

Sixteen patients with a range of 4–10 metastases each, for a total of 112 metastases, were included in this study. The patient's age ranged from 36 to 81 years old and consisted of the following primary cancers: renal cell carcinoma, esophageal, oropharyngeal, melanoma, breast, colon, and non-small cell lung carcinoma (adenocarcinoma and large cell). Five of the 16 patients did receive prior radiation treatment: SRS alone, WBRT alone, or both SRS and WBRT. The target volumes and prescribed doses (Gy) are detailed in Table 1 for each of the 16 patients.

**Table 1:** Target size and prescribed dose per metastasis for the 16 patients included in this SRS study comparison.

**Patient#1**	**Target**	**A**	**B**	**C**	**D**						
	Target volume (cc)	0.97	0.37	4.59	9.63						
	Prescription (Gy)	21	21	18	15						
Patient #2	**Target**	**A**	**B**	**C**	**D**	**E**					
	Target volume (cc)	1.41	0.25	0.52	0.31	0.95					
	Prescription (Gy)	21	21	21	21	21					
Patient #3	**Target**	**A**	**B**	**C**	**D**	**E**					
	Target volume (cc)	0.33	0.65	0.51	0.18	0.17					
	Prescription (Gy)	24	21	24	24	24					
Patient #4	**Target**	**A**	**B**	**C**	**D**	**E**					
	Target volume (cc)	2.73	0.40	0.29	0.30	0.20					
	Prescription (Gy)	15	21	21	21	21					
Patient #5	**Target**	**A**	**B**	**C**	**D**	**E**	F				
	Target volume (cc)	3.54	0.18	0.23	2.26	0.48	0.15				
	Prescription (Gy)	15	18	18	15	18	18				
Patient #6	**Target**	**A**	**B**	**C**	**D**	**E**	F				
	Target volume (cc)	3.4	8.01	0.21	1.52	0.58	2.78				
	Prescription (Gy)	21	15	21	21	21	16				
Patient #7	**Target**	**A**	**B**	**C**	**D**	**E**	F				
	Target volume (cc)	7.12	6.48	0.55	0.37	6.45	2.49				
	Prescription (Gy)	15	18	24	24	15	21				
Patient #8	**Target**	**A**	**B**	**C**	**D**	**E**	F	G			
	Target volume (cc)	0.13	0.14	0.21	0.12	7.98	0.23	0.15			
	Prescription (Gy)	21	21	21	21	15	21	21			
Patient #9	**Target**	**A**	**B**	**C**	**D**	**E**	F	G			
	Target volume (cc)	1.93	4.08	0.31	2.05	8.36	1.62	1.22			
	Prescription (Gy)	21	18	21	21	15	21	21			
Patient #10	**Target**	**A**	**B**	**C**	**D**	**E**	F	G			
	Target volume (cc)	0.69	0.71	0.98	0.55	5.02	0.36	1.3			
	Prescription (Gy)	21	21	21	21	15	21	21			
Patient #11	**Target**	**A**	**B**	**C**	**D**	**E**	F	G	H		
	Target volume (cc)	1.7	0.31	0.15	0.31	0.21	2.45	0.76	0.72		
	Prescription (Gy)	21	21	21	21	21	21	21	21		
Patient #12	**Target**	**A**	**B**	**C**	**D**	**E**	F	G	H		
	Target volume (cc)	2.17	0.44	0.25	0.10	3.62	0.38	0.31	0.32		
	Prescription (Gy)	21	21	21	21	18	21	21	21		
Patient #13	**Target**	**A**	**B**	**C**	**D**	**E**	F	G	H		
	Target volume (cc)	0.91	8.62	0.21	0.33	0.26	0.12	4.21	0.23		
	Prescription (Gy)	21	15	21	21	21	21	18	21		
Patient #14	**Target**	**A**	**B**	**C**	**D**	**E**	F	G	H	I	
	Target volume (cc)	0.16	0.11	0.19	0.94	0.26	5.05	0.38	3.56	0.50	
	Prescription (Gy)	21	21	21	21	21	18	21	18	21	
Patient #15	**Target**	**A**	**B**	**C**	**D**	**E**	F	G	H	I	J
	Target volume (cc)	7.67	0.61	0.20	0.17	0.17	0.47	0.41	0.24	0.21	0.35
	Prescription (Gy)	15	21	21	21	21	21	21	21	21	21
Patient #16	**Target**	**A**	**B**	**C**	**D**	**E**	F	G	H	I	J
	Target volume (cc)	0.22	0.10	0.59	1.58	0.25	1.64	0.91	0.37	0.30	0.40
	Prescription (Gy)	18	18	18	18	18	20	20	20	20	20

Details on each of the SRS modalities utilized in this comparison study are described as follows. The most up to date commercially available product is the Leksell GammaKnife Icon (Elekta, Stockholm, Sweden), containing 192 ^60^Co sources and 4, 8, and 16 mm cone collimator options, which is an upgrade of the Perfexion unit, in that it allows frameless treatments with the addition of on-board cone-beam computed tomography (CBCT) imaging and a real-time motion tracking device. BrainLab Elements™ v1.5 is a commercial treatment planning system that automatically optimizes a dedicated group of dynamic conformal arcs to treat each of the lesions within the brain (via a single arc or a composition of multiple arcs) with a single common isocenter. Volumetric arc therapy enables intensity-modulated dose delivery via varying MLC positions and dose rate, simultaneous to varying gantry rotation speed, thus significantly increasing the degrees of freedom for the optimization algorithm. There is no physical difference in terms of the delivery for conventional VMAT vs. HyperArc. The major difference lies on the planning side for HyperArc, where the software assists the user by automatically selecting an optimal mono-isocenter, collimator angles, and non-coplanar arc setup with the intent of delivering the most conformal plan while minimizing low dose spillage into the surrounding normal brain structures. With conventional VMAT optimization, the planner is responsible for selecting and manipulating all of these variables.

For every patient, a treatment plan was generated according to each of the four SRS techniques: GammaKnife, Elements, Manual-VMAT, and HyperArc-VMAT. Note, all patients were treated clinically with Elements and all other modalities were retrospectively planned for comparison in this study. A total of three different planners were included in this study. A single planner created all of the treatment plans across all patients per specified SRS modality to remove planner variability within each SRS modality. A single SRS planner with 8–10 years of experience generated all of the Elements plans used to treat the 16 patients in this study. A second SRS planner with 1–3 years of experience generated all GammaKnife plans and one set of Manual-VMAT_A_ plans across all patients. Finally, a third SRS planner with 3–5 years of experience generated all Manual-VMAT_B_ plans for the 16 patients. All HyperArc-VMAT plans were generated by the same planner for Manual-VMAT_B_ but after all manual plans were done, i.e., Manual-VMAT_B_ plans were not influenced by HyperArc plans. An additional Manual-VMAT plan was created for every patient following another institution's planning standard, in order to also evaluate the potential differences that may arise between two different treatment planner's styles. The difference in planning techniques between the two VMAT plans are summarized as follows: for VMAT_A_ an upper and lower constraint was used for all targets, and no aggressive objective on low dose spread was applied, whereas VMAT_B_ only applied lower constraints to target volumes but with additional objectives to control low dose spread.

Beam arrangements for the Elements plans were selected from a set of six predefined templates with a range of 5–6 couch angles with 28, 32, 35, or 40 degrees of separation. The gantry angles are initially set to default values ranging from 10° to 170° for couch angles of 0°-90° and 190°-350° for couch angles of 270°-360°. However, these are automatically adjusted during optimization. The plan resulting in the highest conformity from these six templates was selected for treatment and subsequent use in this comparison study. Manual-VMAT_A_ plans consistently used 4 gantry arcs across all patients: 1 full 180° arc without a couch kick and 3 partial arcs ranging from 100° to 160° (depending on isocenter location to avoid entering through the eyes) each separated by 45° couch kicks at 45°, 90°, and 315° couch angles. Manual VMAT_B_ plans also used 4 gantry arcs across all patients: 1 full 180° arc without a couch kick and 3 partial arcs of 170° (with no beamlets going through the eyes) also separated by 45° couch kicks at 45°, 90°, and 315° couch angles. HyperArc-VMAT plan parameters were consistent for all 16 patients: 1 full 180° arc without a couch kick and 3 partial arcs of 180° separated by 45° couch kicks at 45°, 270°, and 315° couch angles. All linac-based SRS plans were planned with MLC leaf widths corresponding to a Varian TrueBeam (Varian Medical Systems, Palo Alto, CA) fitted with HD-120 MLC.

All linac plans were normalized such that the 100% isodose line covered 99% of the target volume. The GammaKnife plans were normalized with the same goal of covering 99% of the target volume with the prescription dose. This resulted in a range of 49–73% prescribed isodose lines with a median of 54%. All of the plan doses were imported into the same treatment planning system platform and version of Varian Eclipse (Varian Medical Systems, Palo Alto, CA) for dosimetric evaluation at a calculation grid size of 1 mm. Note, target normalization was entirely performed in each plan's native treatment planning system and no differences in target coverage were discovered after importing into Eclipse during dosimetric evaluation. The target volume metastasis for all patients in this study was defined as the planning target volume (PTV), already incorporating setup margins. All of the extracted and calculated dosimetric parameters described below are compared equivalently across all SRS techniques in terms of PTV. Thus, there are no inherent biases in comparing conformity indices for GTV vs. PTV when comparing GammaKnife vs. linac-based SRS.

The following dosimetric parameters were extracted per patient target volume across all SRS treatment plans: RTOG conformity index (CI-RTOG) defined as the ratio of the 100% isodose volume to the target volume; Paddick conformity index (CI-Paddick) defined as the ratio of the square of the volume of the target enclosed by the 100% isodose volume to the multiplication of the target volume with the 100% isodose volume; Gradient Index (GI) defined as the ratio of the 50% isodose volume to the 100% isodose volume; and the volume of 12Gy delivered to the surrounding brain tissue contributed only from that individual target (V_12Gy_) and the volume of 12Gy delivered to the surrounding brain tissue per individual target after subtracting that individual target volume (V_12Gy_-TV). Additionally, the following dosimetric parameters were extracted per patient across pertinent organs-at-risk (OARs): the total volume of brain receiving 12Gy, 6Gy, and 3Gy (V_12Gy_, V_6Gy_, V_3Gy_) the mean dose to the brain excluding the targets (Brain mean dose), the maximum dose to the brainstem (D_max_ Brainstem), maximum dose to the left eye and optic nerve (D_max_ L Eye and D_max_ L ON), maximum dose to the right eye and optic nerve (D_max_ R Eye and D_max_ R ON), and maximum dose to the optic chiasm (D_max_ OC). Lastly, the total treatment time for each plan was also extracted for comparison (linac plans times were calculated assuming a dose rate of 1,400 MU/min).

Statistical evaluation of the extracted parameters was performed with JMP Pro v14 (SAS, Cary, NC). The Wilcoxon signed rank test was applied in the format of matched pairs to compare each of the plans against each other per extracted dosimetric parameter. Differences were found to be statistically significant with *p* < 0.05.

## Results

**Figure 1:**
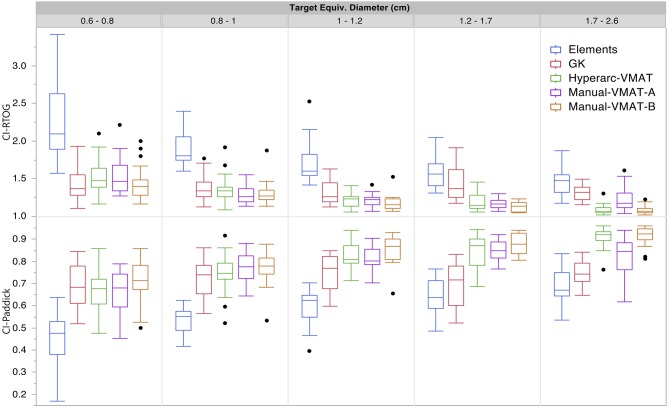
Conformity index results for both RTOG and Paddick definitions displayed as box plots per SRS plan type, divided into five separate target size diameter bins.

**Figure 2:**
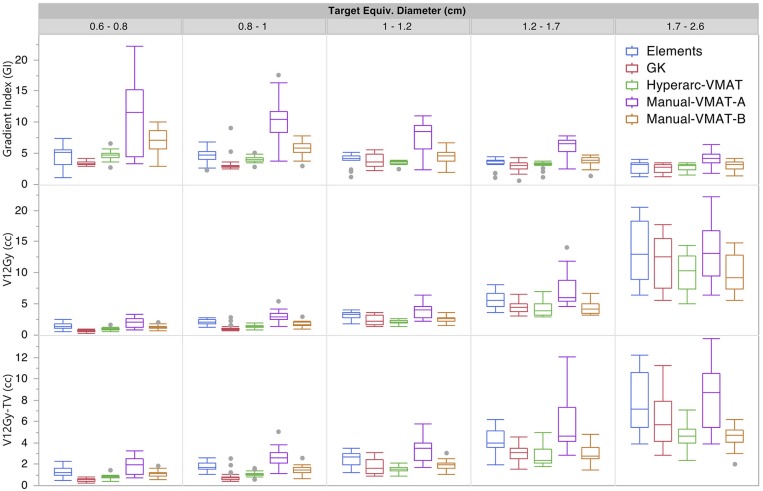
Gradient Index (GI), V12_Gy_ per target (defined as the volume of 12Gy delivered to the surrounding brain tissue contributed only from that individual target), and V12_Gy_-TV(defined as the total volume of brain receiving 12Gy per target excluding the target volume) results displayed as box plots per SRS plan type, divided into five separate target size diameter bins.

Figure 1 graphically compares both types of conformity indices across all five SRS plans grouped according to target size. It is evident that for very small target sizes (<1 cm), GammaKnife, HyperArc-VMAT and both Manual-VMAT plans perform similarly well across both conformity indices. All are superior to the Elements conformity results. However, for target size diameters above 1 cm, HyperArc-VMAT and both Manual-VMAT plans result in superior conformity as compared to GammaKnife and Elements. Figure 2 also graphically divides the results per target bin size for GI and both V_12Gy_ dose metrics. The GI results show that GammaKnife is superior amongst small target diameters (<1 cm), but above that GI is similar amongst all techniques with the exception of VMAT_A_ with the largest range. Amongst the two V12_Gy_ parameters, it is apparent that HyperArc-VMAT is slightly inferior compared to GammaKnife for the small targets (<1 cm) and even outperforms GammaKnife for large targets above 1 cm in diameter. When comparing total V_12Gy_ per patient, i.e., combining all per target V_12Gy_, HyperArc-VMAT is slightly lower than GammaKnife by a median difference of 1.3 cc, which is statistically significant but clinically equivalent. Not surprisingly, the data in Figure 2 demonstrates an increase in both V_12Gy_ metrics as the target size increases. Also noteworthy are the widely variable results between the two Manual-VMAT planning techniques, where VMAT_B_ consistently provides lower V_12Gy_ and V_12Gy_-TV volumes of the brain than VMAT_A_. Yet, neither Manual-VMAT plan performed as well as the HyperArc-VMAT amongst these parameters.

**Figure 3:**
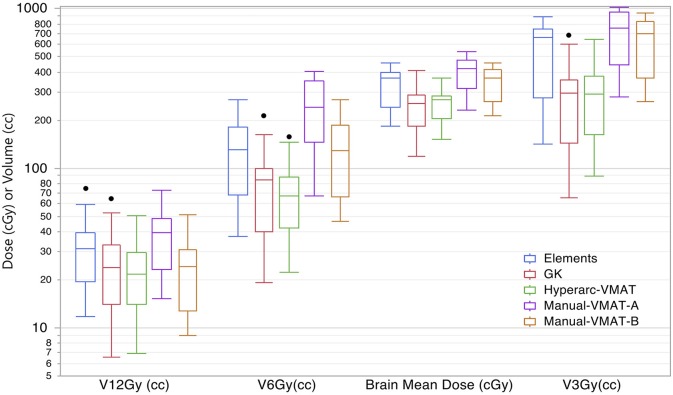
Box plot results per SRS plan type for the following dosimetric parameters across all patients: the total volume of brain receiving 12Gy, 6Gy, and 3Gy (V_12Gy_, V_6Gy_, V_3Gy_) and the mean dose to the brain excluding the targets (Brain mean dose).

Displaying all of the data together, rather than dividing by target bin size, Figure 3 displays the trends observed amongst the remaining extracted parameters representative of low dose spread: brain mean dose, V_12Gy_, V_6Gy_, and V_3Gy_. Here it is again evident that HyperArc-VMAT is comparable with GammaKnife in terms of low dose spillage into the brain, when looking at the entire dataset of target sizes. Elements performs similarly to the Manual-VMAT plans, but inferior to GammaKnife and HyperArc-VMAT in this aspect. The visually evident differences amongst the plans in Figures 1, 3 are further detailed in Table 2, which lists the median difference as well as the Wilcoxon signed rank results per extracted parameter for every potential matched pair of plan comparisons amongst the five options. The median differences are displayed as a result of the row plan subtracted from the column-listed plan. Because a majority of the table displays statistically significant differences with *p* < 0.05, the only 6 (of a total of 70) non-significant *p*-values were instead bolded and underlined in the table to stand out. The purpose of this table was to serve as a more detailed reference of the magnitude of the differences when looking at two specific SRS plans per extracted dosimetric parameter.

**Table 2:** Median differences and Wilcoxon signed rank statistics for matched pair plan comparisons for conformity indices, gradient index, mean brain dose, and volume of the brain receiving 12Gy, 6Gy, and 3Gy.

**Paired Median Differences and Statistical Significance**					
		**Gamma Knife**	**HyperArc-VMAT**	**Manual-VMAT_**A**_**	**Manual-VMAT_**B**_**
Elements	CI-RTOG	−0.35	−0.46	−0.46	−0.50
	CI-Paddick	0.15	0.22	0.20	0.25
	Gradient index	−0.75	−0.33	3.58	0.73
	Brain mean dose (cGy)	−82	−73	74	**19 (*****p*** **=** **0.14)**
	V_12Gy_ (cc)	−5.5	−7.8	6.4	−7.0
	V_6Gy_ (cc)	−45	−52	98	**5.8 (*****p*** **=** **0.4)**
	V_3Gy_ (cc)	−210	−210	160	100
GammaKnife	CI-RTOG		−0.11	−0.077	−0.15
	CI-Paddick		0.085	0.058	0.11
	Gradient index		0.70	4.14	1.36
	Brain mean dose (cGy)		18	140	100
	V_12Gy_ (cc)		−1.3	13.0	**−0.12 (*****p*** **=** **0.98)**
	V_6Gy_ (cc)		−3.7	150	56
	V_3Gy_ (cc)		**36 (*****p*** **=** **0.07)**	370	270
HyperArc-VMAT	CI-RTOG			**0.0 (*****p*** **=** **0.9)**	−0.048
	CI-Paddick			**0.0 (*****p*** **=** **0.3)**	0.026
	Gradient index			4.0	0.95
	Brain mean dose (cGy)			140	90.0
	V_12Gy_ (cc)			17	1.9
	V_6Gy_ (cc)			160	65
	V_3Gy_ (cc)			390	310
Manual-VMAT_A_	CI-RTOG				−0.041
	CI-Paddick				0.030
	Gradient index				−3.0
	Brain mean dose (cGy)				−55
	V_12Gy_ (cc)				−15
	V_6Gy_ (cc)				−92
	V_3Gy_ (cc)				−67

*Median differences are a result of column plan—row plan. All non-bolded cells have statistically significant difference (p < 0.05). P-values that were not statistically significant are bolded and underlined. Blank areas of the table indicate repeat or non-sensical plan combinations*.

**Figure 4:**
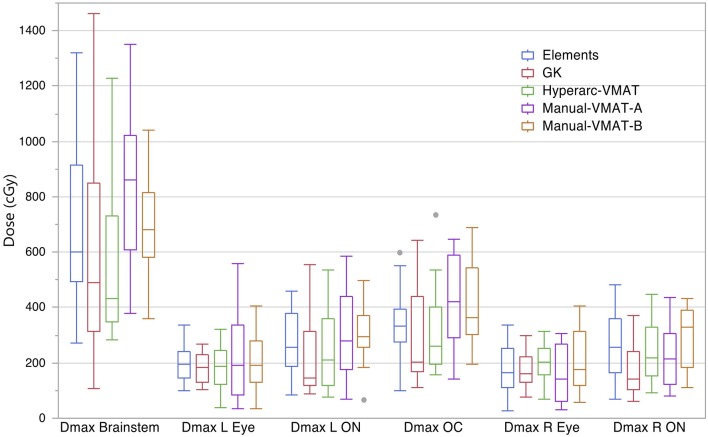
Box plot results per SRS plan type for the following dosimetric parameters across all patients: the maximum dose to the brainstem (D_max_ Brainstem), maximum dose to the left eye and optic nerve (D_max_ L Eye and D_max_ L ON), maximum dose to the right eye and optic nerve (D_max_ R Eye and D_max_ R ON), and maximum dose to the optic chiasm (D_max_ OC).

Figure 4 compares the plan results for all of the studied OARs: maximum dose to the brainstem, optic chiasm left and right eyes, left and right optic nerves. It is important to note that each of the plans satisfied normal tissue constraints amongst all of the patients. Overall, not many patterns nor striking differences between the SRS techniques were observed when it came to sparing OARs and in general they all performed similarly well. The large range observed in D_max_ Brainstem for GammaKnife planning is a result of target location coupled with source geometry and an inability to optimize the beam's trajectory as is possible with Elements and VMAT treatment planning software.

**Figure 5:**
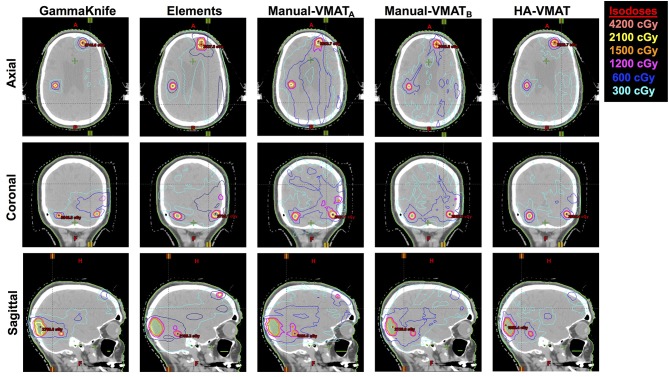
Axial, coronal, and sagittal cuts of patient #15 selected to visually demonstrate differences in dose distribution among the five different SRS plans.

As a visual comparison of the dosimetric results, Figure 5 displays axial, coronal, and sagittal views of the five different SRS plans per patient case #15 with a total of 10 metastases. This patient was selected due to the presence of multiple small metastases as well as a larger, more irregularly shaped target volume, all treated within the same plan. The slice locations were selected so as to show case as many of the treated metastases as possible.

Lastly, treatment delivery times listed in Table 3 were extracted from the GammaKnife treatment plans and approximately calculated for the Elements and Manual-VMAT plans based on the total MUs required (since the dose rate and gantry rotation speed can vary), assuming a dose rate of 1,400 MU/min with 6X flattening-filter-free energy. Unsurprisingly, GammaKnife plans took hours longer to deliver than any linac-based radiosurgery plan. Elements and Manual-VMAT_A_ had similar beam-on times, but HyperArc-VMAT and Manual-VMAT_B_ were longer for almost every single case. The higher MU is a result of the increased modulation, which often happens when more stringent constraints are applied during the optimization process. This is consistent with the brain V_12Gy_ results exhibited in Figure 2 and the mean differences listed in Table 2, where HyperArc-VMAT and Manual-VMAT_B_ result in the least low dose spillage across all target size groups.

**Table 3:** Comparison of total beam-on time in minutes per patient SRS plan, assuming a dose rate of 1,400 MU/min for the linac-based options.

**Beam-On Time (min)**				
**Gamma Knife**	**Elements**	**HyperArc-VMAT**	**Manual-VMAT_**A**_**	**Manual-VMAT_**B**_**
158	4.09	5.95	3.80	9.99
149	5.27	7.08	3.14	7.28
97.7	5.63	4.49	4.71	9.13
182	5.77	6.53	7.32	9.61
128	5.96	6.41	4.85	7.47
156	5.39	5.87	4.13	7.24
143	4.61	5.35	4.95	8.41
162	5.24	6.86	4.74	8.29
160	4.75	6.73	4.29	7.96
166	6.08	6.53	6.08	7.60
111	3.17	4.34	3.55	6.52
116	3.88	6.84	3.43	7.48
146	3.96	5.16	3.05	6.36
111	4.22	6.07	4.04	7.83
113	4.27	7.39	4.85	10.1
141	4.52	5.60	4.82	7.06
**140.0** **±** **24.4**	**4.80** **±** **0.849**	**6.08** **±** **0.897**	**4.48** **±** **1.09**	**8.02** **±** **1.16**

*The last row lists the mean ± standard deviation across all 16 patients, per SRS treatment technique*.

## Discussions

The overall findings of this comparison study have demonstrated that as would be expected, all of the commercially available options for SRS are able to achieve acceptable conformality and OAR dose sparing limits. However, looking more closely at each dosimetric parameter has revealed interesting information. While it was not surprising to find the improved conformity results of the linac-based SRS techniques over GammaKnife for larger and more irregular volumes (due to the more advanced inverse optimization features as well as the ability of MLC shaping), it was certainly unexpected to see HyperArc-VMAT be able to compete with GammaKnife in terms of V12_Gy_. Also expected was GammaKnife's outperformance amongst GI for small targets. However, for the larger target sizes, GammaKnife resulted in similar GIs to HyperArc-VMAT and Manual-VMAT_B_. This information coupled with the results from Table 3 of total beam-on times of minutes vs. hours, suggests that linac-VMAT radiosurgery is a valuable contender to GammaKnife for patients seeking treatment of multiple brain metastases, particularly for large and irregularly-shaped target volumes.

Another rather interesting find was the large deviation seen in the results between the Manual-VMAT_A_ and VMAT_B_ plans, where the optimization objective setting was the main difference between the two techniques, with one having applied upper constraints (VMAT_A_) and the other avoiding upper constraints entirely (VMAT_B_) but with a more stringent control on low dose spread. VMAT_B_ outperformed VMAT_A_ across basically all of the studied parameters: CI, GI, V12_Gy_, V6_Gy_, OAR doses, etc., but all essentially at the cost of longer beam-on times. This large variation in plan quality indicated that the quality of care using VMAT for the treatment of multiple brain metastases is largely dependent on planner experience and institutional standards. Thus, in order to improve the standardization of quality of care, planning procedures and optimization objective settings need to be carefully standardized across our community even at this level of detail.

Furthermore, it can be seen from the results that even though Manual-VMAT_B_ had in general the longest beam-on time, i.e., highest modulation complexity, its plan quality was still mostly inferior compared to HyperArc-VMAT. This indicates that the objective settings used in VMAT_B_ are suboptimal and do not provide as good of a balance (relative to HyperArc-VMAT) between modulation complexity and plan quality. To this end, HyperArc-VMAT could help improve both the optimization efficiency and plan quality standardization for SRS treatment of multiple brain metastases using a VMAT delivery technique.

**Figure 6:**
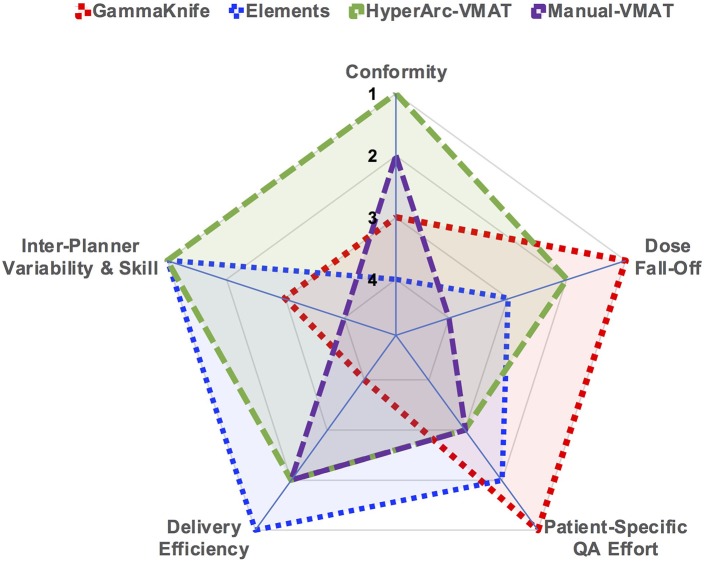
Spider plot graphically comparing the studied SRS techniques across the different categories of dosimetry and efficiency. SRS treatment modalities were ranked relative to each other per specified category on a scale of 1 through 4.

As a quick and straightforward summary of our findings, a spider plot was generated in Figure 6 to serve as a qualitative description of the data. The categories spanned not only dosimetric results, but also considered efficiency and skill in terms of staff and time resources required: conformity, low dose fall-off, inter-planner variability and skill, delivery efficiency, and patient-specific QA effort. Each of the SRS techniques (GammaKnife, Elements, HyperArc-VMAT, and Manual-VMAT) was ranked relative to each other according to the specific category item. Across the different target size bins, Figure 1 demonstrated that HyperArc-VMAT resulted in comparable or superior CI amongst the SRS techniques, thus earning a ranking of 1. GammaKnife had excellent conformity at the smaller target size bins, but that deteriorated with increasing size (compared to VMAT), thus earning it a ranking of 3, after VMAT with a rank of 2. Elements was consistently inferior to the other SRS modalities in terms of CI and thus was ranked last at 4. Regarding the category of dose fall-off, GammaKnife was consistently superior according to Figures 2, 3, thus it was ranked the highest (1), followed by HyperArc-VMAT (2), Elements (3) and then Manual-VMAT (4), due to the dependence on planning strategy and skill. In terms of required planning skill and inter-planner variability, Elements and HyperArc-VMAT are less dependent on this aspect, in that all of the programmed presets only require minimal planner interaction, thus earning both a ranking of 1. GammaKnife would then rank lower (at 3), given that each target is typically forward-planned by the user. (Note however that the forward-planning of multiple metastases in GammaKnife allows the user to fine-tune the coverage of each target, whereas in VMAT planning the software only allows normalization to a single target at the highest dose level when prescribing different doses to different size metastases.) Manual-VMAT ranked the lowest at 4, due to the potential for greatest variability amongst different planners with the large degree of customizable plan settings (compared to GammaKnife), which can result in varying plan quality as seen in plans A vs. B. Table 3 displays the beam-on time and thus the delivery efficiency are straightforward in this respect: Elements had the lowest average beam-on time (rank = 1), followed by comparable beam-on times of HyperArc-VMAT and Manual-VMAT (both ranked at 2), and GammaKnife coming in last (rank 4) with the longest beam-on times. Furthermore, GammaKnife treatment requires the presence of an authorized medical physicist as well as a physician trained in emergency procedures for the entirety of the treatment, which may pose an additional burden on staff resources (as compared with linac-based radiosurgery). Lastly, when it comes to required patient-specific QA, GammaKnife does not require any and thus would be ranked the highest at 1, followed by Elements ranking at 2 (whether to perform dose verification for 3D-DCA SRS plans varies according to institutional policies) and then both VMAT techniques (all ranked at 3) which require additional resources i.e., physics staff to perform the time-consuming QA, involving plan preparation, device setup, beam delivery and plan analysis. The overall purpose of Figure 6 is to allow the reader to qualitatively evaluate the differences in focus amongst the SRS techniques per category of interest, in the context of multiple metastases treatment.

Another practical aspect to consider when interpreting the differences seen in the results is the accuracy and precision of these treatment machines and how truly capable they are to deliver exactly what is displayed to the user in the treatment planning software. Inevitably, uncertainties exist throughout the entire treatment process, from simulation to on-board imaging and patient setup, all the way through to radiation delivery. Although it is beyond the scope of this paper, it is important to be aware of the potential geometric uncertainties present not only from the hardware (imaging and radiation isocenter coincidence, gantry rotation and sag, couch positional accuracy, MLC positional accuracy, etc.), but in the patient immobilization (frameless mask treatments for linac and GammaKnife) aspect as well, which can alter the expected conformity indices as calculated by the planning software. This type of data analysis will be the goal of our future studies.

Upon evaluation of the dosimetric and logistical differences of these currently available SRS treatment techniques, the question arises whether any of these differences actually have a clinically tangible impact. The clinical implications of the disparities in the low dose spillage or the conformity indices, in terms of local control or quality of life, is a much more vast and complicated discussion that ultimately is very difficult to determine. It would require multi-institutional prospective clinical trials with long term follow-up, which sadly may be rather difficult to obtain, given the average length of survival of patients with multiple brain metastases. However, for the purposes of this comparison study, we have analyzed and presented the data in such a manner as to provide the community with a tool for selecting an SRS modality for a specific patient scenario when more than one option is available, or even for the case of selecting which type of SRS modality fits best within one's clinical needs based on their specific patient population.

## Conclusions

HyperArc-VMAT and Manual-VMAT plans resulted in superior CI when compared with GammaKnife and Elements for target diameters > 1 cm in size, albeit at the expense of more MUs (relative to Elements). For targets < 1 cm, GammaKnife, HyperArc-VMAT and both Manual-VMAT plans achieved similar CI, but still all superior to Elements. In the smaller target size bins, GammaKnife resulted in superior GI. In terms of low dose spread into the brain, HyperArc-VMAT achieved comparable (target size < 1 cm) or slightly better V12_Gy_ values as GammaKnife (target size > 1 cm). All five SRS plans were able to meet the surrounding normal tissue limits, and overall resulted in similar doses to the pertinent OARs. Beam-on times were hours longer for GammaKnife vs. each of the linac-based SRS plans, with VMAT_A_ and Elements resulting in shorter times relative to VMAT_B_ and HyperArc-VMAT. Manual-VMAT plan quality varied greatly between the two institutional planning strategies employed.

In summary, this study demonstrated that HyperArc-VMAT is capable of achieving similar or slightly better low dose spread into the brain as GammaKnife, while maintaining excellent conformity as well as minimizing inter-planner variability and beam-on time for patients seeking treatment of multiple metastases. GammaKnife remains superior in terms of gradient index and eliminates the need for patient-specific QA. Elements strengths include delivery/QA efficiency and inter-planner consistency due to automated optimization of pre-defined templates. Manual-VMAT is subject to larger inter-planner variability as compared to HyperArc-VMAT.

## Data Availability

All datasets generated for this study are included in the manuscript and/or the supplementary files.

## Ethics Statement

This study was carried out in accordance with the recommendations of Thomas Jefferson University, Internal Review Board. The protocol was approved by Thomas Jefferson University IRB (#18D.480) granting a waiver of informed consent.

## Author Contributions

IV, TL, and HL designed the study, performed experiments, and data analysis. IV drafted the manuscript with assistance from TL. HL assisted with data collection. MA-B, LD, JL, KN, WS, BT, NY, YY, and WZ all contributed substantially to the concept, design of the study and preparation of the manuscript.

### Conflict of Interest Statement

HL received grant funding unrelated to this work from Varian Medical Systems and BrainLab. TL received personal fees from Varian Medical Systems unrelated to this work. The remaining authors declare that the research was conducted in the absence of any commercial or financial relationships that could be construed as a potential conflict of interest.
